# Covid-19 Measures in Switzerland—Considerations from a Practice Perspective

**DOI:** 10.1007/s40804-023-00279-1

**Published:** 2023-03-15

**Authors:** Luca Jagmetti

**Affiliations:** Attorney-at-law, partner with Bär & Karrer AG, Zürich, Switzerland, Dr. iur. LL.M., Co-Head of the Practice Group Turnaround, Reorganization and Insolvency of Bär & Karrer AG, Brandschenkestrasse 90, 8002 Zürich, Switzerland

**Keywords:** Covid-19, Fiscal measures, Short-time work, Covid loans, Restructuring

## Abstract

This paper offers some observations from a practitioner perspective on Swiss measures to support businesses during the Covid-19 pandemic, as a complement to the paper ‘Governmental measures in Switzerland against mass bankruptcies during the Covid-19 pandemic’ by Rodriguez and Ulli in this volume. A brief overview of the main fiscal and non-fiscal measures is followed by analysis of the reasons for non-use of a major non-fiscal measure (a new moratorium), and some suggestions as to the lessons that can be learned from this for the design of analogous relief policies in future crises.

## Introduction

As from March 2020, the Swiss federal government implemented an array of Covid-19 measures based on emergency competences in the Constitution. Some measures were specifically designed for certain industries (e.g., the travel sector, or support for sports/cultural institutions), but the majority were of a general nature, applying across industries. In this paper, I focus on the latter type of intervention, i.e., non-sector specific measures, and seek to address the following questions in this regard:

- Why were some of these measures widely used and—measured in terms of popularity—successful, while others were hardly applied?
- What are the lessons that we can learn from experiences with these measures for the design and implementation of analogous measures in a future crisis?

I do not consider the—political and debatable—question of whether the goals pursued by the implemented measures were sensible in all instances.

The article begins with a brief overview of the main fiscal and non-fiscal measures for supporting distressed businesses in Switzerland (Sect. 2), before turning to the evidence of (non-)use of these measures (Sect. 3), and the lessons that can be learned from this (Sect. 4).

## Overview of Swiss Covid-19 Measures

In the following, a condensed overview is provided of the main fiscal measures (Sect. 2.1) and the restructuring law-specific measures (Sect. 2.2) implemented in Switzerland during the Covid-19 pandemic.

### Fiscal Measures

#### Short-Time Work (*Kurzarbeit*)

From a practical perspective, the so-called short-time work (*Kurzarbeit*) was probably *the* most important governmental support measure in Switzerland. It was not a Covid-specific tool, but already existed as part of the Swiss unemployment insurance legislation^1^ and can generally be applied by enterprises in distress situations. Its goal is to avoid layoffs in situations where the reduction in work is expected to be of limited duration. Enterprises can—with the consent of the affected employees—temporarily reduce the working hours of their employees with a corresponding reduction in wages. Provided the respective conditions are met, the Swiss Unemployment Insurance scheme will cover up to 80% of the loss of income attributable to the reduction in working hours. In other words, the employees agree to reduce working time, but continue to receive 80% of the salary for the time they are not working, which is borne by the Swiss Unemployment Insurance scheme. So, the employer only incurs the cost of the time that employees are actually working.

During the pandemic, the Swiss Federal Council lowered, by its Covid-19 Ordinance on Unemployment Insurance,^2^ the hurdles for access to short-time work compensation and simplified the application processes for enterprises, thereby expediting the handling and payout by the unemployment insurance.

#### Covid Loans

As in many other jurisdictions, Switzerland also implemented a Covid loan programme to provide rapid and non-bureaucratic access to liquidity for enterprises with a turnover of no more than CHF 500 million in 2019, in an amount of up to 10% of their pre-pandemic turnover. Under the widely used programme, commercial banks granted two types of bridge loans to enterprises, which were backed by the Swiss government:^3^

- ‘Regular Covid-19-Credit’: banks granted loans of up to CHF 500,000 at no interest and on uniform standard terms and conditions for such loans, fully guaranteed by the Swiss Confederation;
- In addition to the Regular Covid-19-Credit, enterprises could take out a ‘Covid-19-Credit Plus’ loan of up to CHF 19.5 million. 85% of such loans carried an interest of 0.5% p.a. and were guaranteed by the Swiss Confederation; the remaining 15% were not state-backed and the interest rate was left to be negotiated between the parties.

These Covid-loans are repayable within 8 years, which can be extended by another 2 years in case of hardship and if the extension is likely to lower the financial risk of the Swiss Confederation.^4^

### Restructuring Law Measures

#### General Stay of Proceedings

The Swiss Debt Enforcement and Bankruptcy Act (‘DEBA’)^5^ gives the federal government the possibility to decree a general stay of debt enforcement proceedings for a certain period of time. At the outset of the pandemic in Switzerland in March 2020, the Federal Council made use of this instrument and ordered a suspension of all debt enforcement acts for almost 3 weeks—which produced a grace period of in total almost 5 weeks (combined with the regular enforcement pause during Easter holidays).^6^

#### Covid Moratorium

The Swiss insolvency regime includes a procedure called composition proceedings (*Nachlassverfahren*; Art. 293 et seq. DEBA), which is similar to—but at the same time has some important differences with—in-court restructuring proceedings of other jurisdictions. Composition proceedings are relatively seldom used in Switzerland^7^ and of less practical significance than, for example, Chap. 11 proceedings under the US Bankruptcy Code.

During the Covid pandemic, the federal government temporarily introduced an additional, new form of composition proceedings for small and medium-sized enterprises through its Covid-19 Ordinance on Insolvency Law.^8^ Upon application to the competent court, companies were granted a payment moratorium of 3 months, which could be extended by another 3 months.^9^ During such period, creditors could not continue debt enforcement steps against the debtor for claims that had arisen prior to the opening of the proceedings, and the debtor was prohibited from (voluntarily) settling such stayed claims (except salaries). The debtor was further prohibited from performing legal acts that (i) impaired the legitimate interests of creditors, or (ii) favoured individual creditors to the detriment of others.^10^ After the lapse of the moratorium, the stayed claims had to be settled. Claims newly arising after opening of the proceedings were not stayed and could be enforced. Such moratorium ‘light’ thus only granted a temporary relief to make payments and could hence be considered useful only in cases where a ‘V-shaped’ business recovery was expected.

#### Suspension of Duty to File for Bankruptcy

Under Swiss law, if a company is over-indebted (liabilities are no longer covered by assets—*Überschuldung*), the board of directors is obliged to file for bankruptcy, unless creditors subordinate claims at a level sufficient to cover the over-indebtedness (or if the company files for composition proceedings) (Art. 725, para. 2, Swiss Code of Obligations—‘CO’). Also, the board may delay a filing for a maximum of 4–6 weeks if it immediately implements restructuring measures and there is a realistic prospect of financial recovery.^11^ While the directors’ obligation to file for bankruptcy is, at first glance, triggered by the balance sheet situation only, bankruptcies are often opened because of illiquidity: under general Swiss accounting law, if continuation of the business activities during the next 12 months is likely not possible (i.e., no longer a going concern; in particular due to lack of liquidity), financial accounting must switch to—usually substantially lower—liquidation values (Art. 958a, para. 2 CO). Therefore, illiquidity often leads to over-indebtedness, which in turn triggers the duty of the board to file for bankruptcy.

In the pandemic, the duty of the board to file for bankruptcy was alleviated in two respects: first, government—guaranteed Covid—loans of up to CHF 500,000 do (still today) not count as liabilities for purposes of assessing whether a company is over-indebted within the meaning of Art. 725, para. 2 CO.^12^ Secondly, the board was relieved from its duty to file for bankruptcy despite over-indebtedness, if the company only incurred the indebtedness in 2020 (i.e., had not yet been over-indebted at 31 December 2019) and there was a prospect (*Aussicht*) that the over-indebtedness could be eliminated by 31 December 2020. The board had to document its decision to abstain from filing for bankruptcy based on the Covid relief, including, e.g., preparing an interim balance sheet and liquidity plans.^13^

## Observations

### Covid Moratorium Hardly Used

Contrary to general expectations, the newly introduced Covid moratorium (Sect. 2.2.2 above) was hardly used in practice.^14^ Regarding the alleviations of the duty of the board of directors to file for bankruptcy (Sect. 2.2.3 above), no data is available on how many companies applied this grace period, given that the associated board resolutions are not publicly available. The fact that the number of bankruptcies did not increase once the Ordinance with the alleviations lapsed^15^ may suggest that no significant number of companies delayed bankruptcy filings based thereon.

The obvious question that arises is why in particular the Covid moratorium was so rarely used. Lacking full data, I offer some conjectures below.

### No Need for Insolvency Measures in View of Fiscal Measures

The first and quite surely most relevant factor for the non-use of the Covid moratorium is that the financial Covid measures described in Sect. 2.1 above rendered the former superfluous: with the short-time work relief, employers could rid themselves of a major source of cash drainage, as they only had to pay for those employees who actually worked. If no work was to be done, no costs were incurred. Remaining liquidity needs of businesses could largely be covered with the Covid loans described in Sect. 2.1.2 above. While the taking out of such loans entailed certain restrictions, e.g., a prohibition on paying out dividends, basically no restrictions applied on an operational level and—after the abandonment of the initial prohibition to use the funds received for new investments^16^—they could be used for the business as desired. Put in somewhat blunter terms, given that the economy was flooded with government money, there was arguably simply no need for further, restructuring-law-specific measures. The situation might have been quite different if financial aid had been less readily available.

With respect to lease agreements—typically another significant source of fixed costs for most small and many medium-sized enterprises—further factors contributed to a reduced liquidity need. First, there was (and is) an ongoing legal debate as to whether an obligation to pay rent subsists at all during the time that the rental object cannot be used due to government-mandated lockdowns. In many instances, landlords and tenants negotiated compromise solutions leading to temporary rent reductions or deferrals. Moreover, during almost 3 months at the beginning of the pandemic, the statutory time period during which a tenant may be in payment default before the landlord can extraordinarily terminate the lease was extended from 30 to 90 days.^17^ Parliament debated a mandatory reduction of rent, but in the end this was not enacted. The threat of this legislation may, however, have fostered agreement for the more amicable solutions between landlords and tenants mentioned above.

### Simplicity of Fiscal Measures

The fiscal measures described above had one thing in common: they were relatively clear to grasp and simple to apply. To obtain short-time work benefits, companies basically just had to complete a short form. The position was similar for Covid loans, which were available on companies filling in a standard application form to obtain credit. This simplicity no doubt facilitated take-up of both schemes.

### Shortcomings of the Covid Moratorium

In contrast to the simplicity of the fiscal measures, understanding the Covid moratorium, its advantages and disadvantages was—by its nature—more complex, and a typical SME would have had to involve an external lawyer for a proper assessment thereof. Further, the moratorium required the involvement of the court. Both of these factors imply a need for upfront cash in a situation where liquidity is scarce by definition.

The Covid moratorium provisions also had disadvantages in their design. The granting of the moratorium had to be published and the debtor had an obligation to actively inform its creditors in writing or by email,^18^ which would have been expected to lead to suppliers demanding pre-payment, thereby increasing liquidity needs even more. More fundamentally, the moratorium only granted relief for existing but not for ongoing obligations, and after at the latest 6 months all claims would become payable again. In the spring/summer of 2020, this was likely too short given the general uncertainty about how the pandemic would develop.

Weighing these downsides against the rather limited benefit of a short stay for existing debt probably, in many cases, resulted in not pursuing the possibility of the Covid moratorium; even more so as bridging a 3–6 months liquidity shortfall was possible—in a much easier way—through the financial aids.

### No Safe Haven

As a further important point, the Covid moratorium as well as the alleviations regarding bankruptcy filing—which were both intended and labelled as relief to debtors—left important uncertainties that undermined their goals to some extent.

As mentioned in Sect. 2.2.2, the Ordinance regarding the Covid moratorium contained a provision according to which the debtor was prohibited from performing legal acts that impaired the legitimate interests of creditors or favoured individual creditors to the detriment of others. It remained vague what such acts, legitimate interests and favouring would have been. If certain creditors are paid during a stay while others are not and ultimately the company goes bankrupt, it can always be argued that the interests of some creditors are impaired. Does this hence mean that during the stay all new obligations must strictly be settled according to their rank and equally within a rank? And once the stay lapses, do all prior claims have to be settled according to rank and pro rata within the last rank? Both seem difficult in practice, would create an administrative burden and a need for outside legal support (creating additional costs), and could thus threaten to imperil the goal of helping debtors. The clause potentially created a route for creditors to later bring personal liability claims against the directors and officers for breach of their (fiduciary) duties (Art. 754 CO) if a restructuring failed.

A similar critique can be levelled at the provision for temporary relief from the obligation to file for bankruptcy. As described in Sect. 2.2.3, abstaining from a bankruptcy filing despite over-indebtedness was only allowed if there was a prospect that the over-indebtedness could be eliminated by 31 December 2020. If the company ultimately went bankrupt nevertheless, it would have been plausible to expect that certain creditors could, with the benefit of hindsight, bring liability claims against the directors, arguing that there was no prospect of eliminating the over-indebtedness by the end of 2020 and the board thus illicitly delayed bankruptcy filing. Remembering the situation in spring/summer 2020, with all uncertainties regarding even short-term developments, next Covid wave(s) and virus variations, it seems extremely difficult for a board of directors at that time to realistically make a prognosis for the period until 31 December 2020.

Applying the two restructuring law measures hence left a risk for personal liability of the acting directors and managers, which of course reduced their attractivity in practice.

## Conclusion

Based on the above observations, the following lessons can be learned from the Covid pandemic with respect to the design of future emergency measures in comparable crisis situations:

- In a first step, the desired *goals* and effects of a measure should be *clearly defined*. What such goals are remains ultimately a political question and must—depending on the circumstances—be agreed upon. It is, for instance, debatable whether keeping in business each and every SME by flooding billions of Swiss francs into the economy is sensible under all circumstances; in particular as it appears that the numbers of bankruptcies now start increasing, which nourishes the suspicion that a large number of bankruptcies have simply been postponed at enormous cost to the public.
- Secondly, once the aim of a measure is defined, its design needs to be structured so as to enable achievement of the defined goal. In order to be effective, measures should be *simple* and easy to apply by their addressees.
- Thirdly, measures that seek to grant relief must actually do so: they need to provide a true safe haven to the acting directors and officers of the company. If a measure is only labelled as relief, but leaves a ‘back door’ open for creditors to later bring personal liability claims, such a measure will likely not be widely used.

In summary, in a crisis situation it is, in my view, preferable to have simple and clear measures with true relief for a short time period rather than compromise measures of longer duration. A good example of such a measure that fulfilled the above criteria were the general stay of debt enforcement actions at the outset of the pandemic. While a longer application would probably have led to severely detrimental effects (as in World War I),^19^ granting a short, but truly comprehensive and simple pause for companies and their managements to regroup and adapt to a completely changed world was probably an important element for the Swiss economy to overcome the crisis.

While the above observations and considerations are neither groundbreaking nor new and apply in other areas as well, it is to be hoped that they are considered by governments and that the experience made during the Covid-19 pandemic so far will improve the handling of future crisis situations.

## Footnotes

1 Art. 31 et seq., Unemployment Insurance Act, https://www.fedlex.admin.ch/eli/cc/1982/2184_2184_2184/de (accessed on 13 February 2023).

2 See Covid-19 Unemployment Insurance Ordinance, https://www.fedlex.admin.ch/eli/cc/2020/169/de (accessed on 13 February 2023).

3 Art. 3 et seq., Covid-19 Solidarity Guarantee Ordinance, https://www.fedlex.admin.ch/eli/cc/2020/194/de (accessed on 13 February 2023).

4 Art. 3, para. 2 Covid-19 Solidarity Guarantee Act, https://www.fedlex.admin.ch/eli/cc/2020/982/de (accessed on 13 February 2023).

5 Resolved upon on 11 April 1889 with the current version effective as of 1 August 2021, https://www.fedlex.admin.ch/eli/cc/11/529_488_529/de (accessed on 13 February 2023).

6 See Eichel and Turtschi (2020), p 1011 et seqq. for more details.

7 Staehelin and Bopp (2020), n. 37.

8 See https://www.fedlex.admin.ch/eli/cc/2020/235/ (accessed on 13 February 2023).

9 Art. 6, para. 1 and Art. 7, para. 1 Covid-19 Ordinance on Insolvency Law.

10 Art. 13, para. 1 Covid-19 Ordinance on Insolvency Law.

11 Wüstiner (2016), Art. 725, n. 40a.

12 Art. 24 Covid-19 Solidarity Guarantee Act, https://www.fedlex.admin.ch/eli/cc/2020/982/de#art_24 (accessed on 13 February 2023).

13 Affolter (2020), p 3; Diem and Ehrsam (2020), n. 40 et seq.

14 Swiss Official Gazette of Commerce, https://www.shab.ch/#!/search/publications?keyword=COVID-19-Stundung (accessed on 13 February 2023); see also Rodriguez and Ulli (2023).

15 See Rodriguez and Ulli (2023).

16 Art. 6, para. 2, let. B Covid-19 Solidarity Guarantee Ordinance, https://www.fedlex.admin.ch/eli/cc/2020/194/de (accessed on 13 February 2023).

17 Art. 2, Covid-19 Leases Ordinance, https://www.fedlex.admin.ch/eli/cc/2020/195/en (accessed on 13 February 2023).

18 Art. 10, para. 2 Covid-19 Ordinance on Insolvency Law.

19 See Rodriguez and Ulli (2023).
